# Anatomical and functional correlates of cystic macular edema in retinitis pigmentosa

**DOI:** 10.1371/journal.pone.0276629

**Published:** 2022-10-21

**Authors:** Adam Ruff, Alangoya Tezel, Tongalp H. Tezel

**Affiliations:** 1 Department of Ophthalmology, Columbia University, Vagelos College of Physicians and Surgeons, New York, NY, United States of America; 2 University of Michigan Medical School, Ann Arbor, MI, United States of America; University Hospitals Cleveland, UNITED STATES

## Abstract

Cystoid macular edema (CME) is a major cause of central visual deterioration in retinitis pigmentosa. The exact reason for CME and its prognostic significance in this patient population is unknown. We seek to find clues to answer these questions by examining the anatomical correlations between retinal cysts and retinal morphometric parameters in a cohort of patients with retinitis pigmentosa and CME. For this reason, 103 patients (196 eyes) with untreated cystoid macular edema (CME) were identified from a pool of 578 genotyped patients with retinitis pigmentosa. Image analyses were conducted using three central horizontal OCT scans of these patients to calculate cross-sectional areas of the retinal nerve fiber layer, outer retinal, inner retinal, cysts, and total retinal areas. Lengths of the ellipsoid zone and outer limiting membrane were also measured. Best-fit curves were derived for analyzing the factors playing a role in the size of the retinal cysts and the patients’ visual acuity. Generalized Estimating Equation and multivariate linear regression analyses were conducted to determine the correlations between visual acuity, morphometric and clinical data, and the significant cyst size and visual acuity determinants. Twenty-five percent of the screened patients (103/578) had CME. Patients with autosomal dominant retinitis pigmentosa had the highest incidence of CME (43.6%, p<0.001) but also had the best visual acuity (20/34±20/30, p = 0.02). The total cyst area was 0.14±0.18 mm^2^. Outer retinal area (B = 0.214; p = 0.008), age (B = -0.003; p<0.001) and retinal nerve fiber area (B = 0.411; p = 0.005) were main determinants of the (r = 0.44; p<0.001) cyst size. Cysts resolved with progressing retinal degeneration. Length of the intact ellipsoid zone (B = -5.16E-5; p<0.001), the inheritance pattern (B = 0.04; p = 0.028) and retinal nerve fiber area (B = 0.751; p<0.001) were the main determinants of visual acuity. In patients with retinitis pigmentosa and cystoid macular edema, retinal nerve fiber layer thickness is associated with decreasing visual acuity and cyst size. This finding suggests that intraretinal cysts may compress retinal axons and cause subsequent visual loss in retinitis pigmentosa.

## Introduction

Retinitis pigmentosa is a group of inherited degenerations characterized by progressive degeneration of the rod and cone photoreceptors. It is the most common hereditary retinal disease and affects 1 in 4000 people [1]. Retinitis pigmentosa is a collection of diseases with overlapping clinical presentations. More than 3000 mutations in 80 genes have been identified so far as the cause of retinitis pigmentosa. These mutations are responsible for defects in phototransduction, rhodopsin cycling, and cell trafficking pathways leading to the progressive death of rod photoreceptors. Initial loss of rods manifests itself with nyctalopia and visual field constriction in the early phases of the disease. In later stages, cone photoreceptor loss leads to severe visual loss and loss of color discrimination [2]. The genetic heterogeneity of retinitis pigmentosa results in its various clinical presentations. Patients with X-linked retinitis pigmentosa constitute 5–15% of the cases and have the worst prognosis compared with patients with autosomal recessive (50–60%) and autosomal dominant (30–40%) forms of the disease [2]. In 20–30% of cases, the expression of mutant retinal proteins in extraocular organs can lead to several systemic findings. These cases are termed the syndromic form of retinitis pigmentosa, and over 30 such clinical syndromes have been described [3].

Since the first mutation in the rhodopsin gene was identified in 1990 [4], attempts have been made to modify or manipulate the expression of mutated genes with several gene treatment techniques. One such effort to correct the biallelic mutation of the RPE65 gene using an AAV vector has been approved by the FDA for treatment [5]. Several studies are in the pipeline to deliver the correct copy of the defective genes or, in the case of autosomal retinitis pigmentosa, perform “gene surgery” using the CRISPR/Cas system to inactivate mutant proteins [6]. However, the heterogeneity of the causative genes limits the development of universal gene therapy for all forms of retinitis pigmentosa. Optogenetics, retinal cell transplantation, retinal prostheses, and medical treatment with neuroprotective, anti-apoptotic, or anti-oxidative agents are all at different development phases to rewire the disrupted retinal retinal circuitry or slow down the retinal degeneration [7].

Several epiphenomena may contribute to the deterioration of central vision in patients with retinitis pigmentosa, including premature lens opacities [8, 9], epiretinal membrane formation [9], CME [9, 10], lamellar [11] and full-thickness macular holes, [12], vitreomacular traction [13], or inner retinal atrophy [14]. The prevalence of CME has been reported to be between 11–70%, depending on the clinical diagnostic method employed [7, 9]. The exact reason patients with retinitis pigmentosa develop CME remains unknown. CME is more prevalent among young patients and patients suffering from autosomal dominant retinitis pigmentosa [9]. Early recognition and treatment of CME are essential, considering the premature deterioration of the peripheral visual field in patients with retinitis pigmentosa and their dependence on central vision [15]. Carbonic anhydrase inhibitors [16], local steroids [17], and anti-VEGF agents [18] have been used for the medical treatment of CME in retinitis pigmentosa with varying efficacies.

CME is ubiquitous in patients with retinitis pigmentosa. However, its natural evaluation, correlation with anatomical changes in different retinal layers, and associations with different clinical and genetic variants of retinitis pigmentosa have not clearly been put forward. Herein, we seek to answer these questions by investigating the anatomical correlations between retinal cysts and retinal morphometric parameters in a cohort of patients with retinitis pigmentosa and CME.

## Material and methods

This retrospective case analysis was approved and monitored by the Institutional Review Board of the Columbia University, Vagelos College of Physicians and Surgeons, and conducted according to the principles of the *Declaration of Helsinki*. At the time of their first presentation, informed consent was obtained from each patient before ocular imaging and genetic analyses. For the minors, their parents’ consent was obtained.

### Study subjects and inclusion criteria

Clinical files and retinal imaging studies of patients with retinitis pigmentosa were screened retrospectively. All the screened patients had genetic workup to identify the causative genes. Only subjects who presented with untreated CME and were followed for more than a year were included in the study. All patients had typical findings of retinitis pigmentosa, such as mid-peripheral bone-spicule pigment clumps, arteriolar attenuation, and waxy disc pallor. Subjects were genotyped using whole exon sequencing (n = 40) or commercially available genetic panels (n = 63).

Strict eligibility criteria were applied to eliminate any confounding factors, including media opacities, high myopia (>6 D), glaucoma, ocular hypertension (IOP>21 mmHg), retinoschisis; vitreomacular traction, retinal surgery or laser, recent (< 4 months) eye surgery, corneal graft, ocular injury, any uveitic entities, age-related macular degeneration, ocular trauma or scars, previous intravitreal steroid injections, and any other retinal vascular or neurodegenerative disease.

Subjects had a complete ocular examination to determine the refraction, best-corrected Snellen visual acuity, applanation tonometry, slit-lamp examination of the anterior segment, and binocular assessment of the posterior segment. CME was treated mainly with systemic or topical carbonic anhydrase inhibitors and periocular steroid injections in a few cases. Clinical data and OCT images obtained before initiation of the treatment were used for the study.

### Image acquisition and analyses

Only patients imaged with spectral-domain Spectralis OCT (Heidelberg Engineering GmbH, Heidelberg, Germany) were included due to the known variability of macular thickness measurements with different OCT devices in retinitis pigmentosa [19]. This OCT device can perform 40,000 A-scans/second with 3.5-μm digital axial resolution. OCT scans were acquired using image alignment eye-tracking software (TruTrack^®^, Heidelberg Engineering GmbH, Heidelberg, Germany). For each eye, 31 horizontal scans covering a 6x6 mm^2^ (20°) area centered at the fovea were obtained using Family Acquisition Module 6.5.2.0. B-scan averaging was set at 16 frames for noise reduction while the eye-tracking system maintained the stability of the image. Only images with a signal strength of >20db were used in analyses to avoid the known interference of CME on OCT signal strength. Foveal location was estimated according to the depth of foveal depression and its relative position to the optic disc. In order to have a broader view of the anatomical changes in the macula, image analyses were conducted using horizontal scans passing through the fovea and 125 μm above and below it.

**Fig 1:**
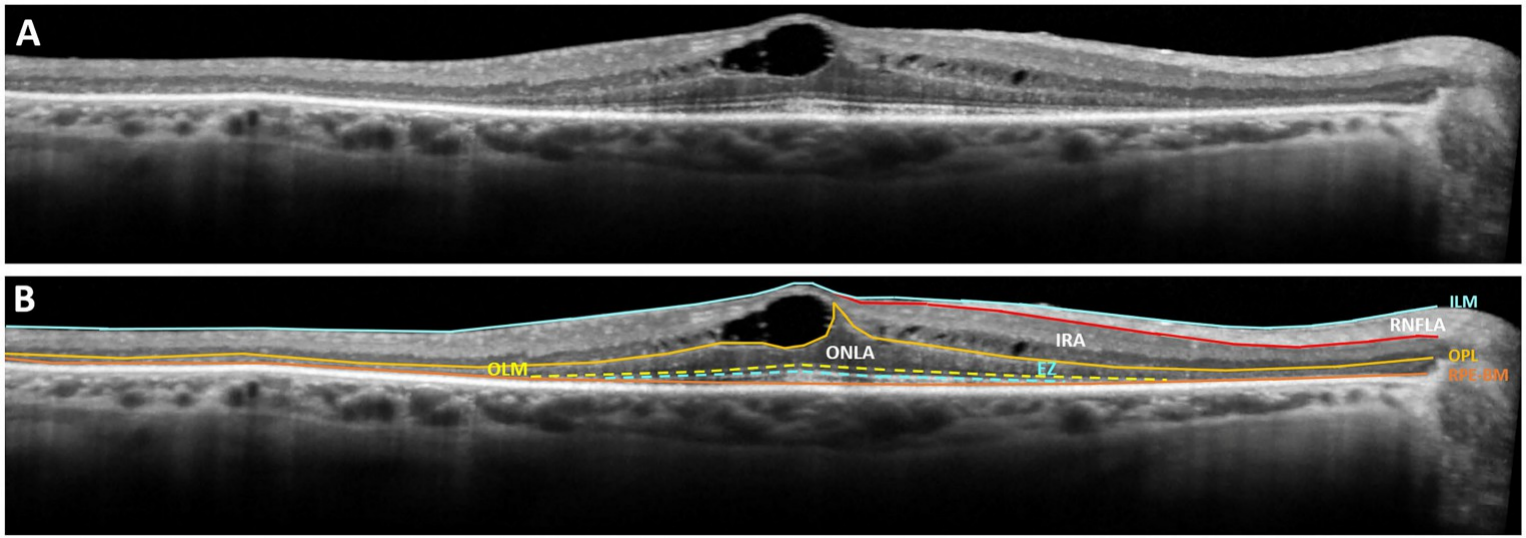
Manual segmentation and morphometric analysis of retinal layers. **(A)** Three horizontal OCT scans passing through the fovea were used for morphometric analyses. **(B)** The boundaries of the inner limiting membrane (straight blue line), the outer border of the retinal nerve fiber layer (red straight line), the mid-outer plexiform layer (orange straight line), and the inner border of the RPE-Bruch’s membrane complex (straight brown line) were traced manually. *“*Retinal Nerve Fiber Layer Area” (RNFLA) was described as the area between the inner border of the internal limiting membrane and the outer border of the retinal nerve fiber layer. The area between the internal limiting membrane and the outer plexiform layer was defined as the *“*Inner Retinal Area” (IRA), including the RNFLA and the cysts. Inner retinal neuronal area (IRNA) was defined as the area occupied by inner retinal neurons and estimated by subtracting the total cyst area and RNFLA from the IRA. The area between the outer plexiform layer and RPE was regarded as the *“*Outer Retinal Area” (ORA). In the presence of cysts in the outer retinal zone, the cyst area was excluded from the outer retinal area calculation. The lengths of the intact OLM (interrupted yellow line) and EZ (blue interrupted line) were also traced and calculated.

Captured OCT scans were manually segmented into three zones by tracing the borders of (1) the inner border of the inner limiting membrane, (2) the outer border of the retinal nerve fiber layer, (3) the mid-outer plexiform layer, and (4) the inner border of the 4^th^ hyperreflective line of the outer retina, which corresponds to Retinal pigment epithelium (RPE)-Bruch’s membrane complex (Fig 1).

The area between the inner border of the internal limiting membrane and the outer border of the retinal nerve fiber layer was accepted as the *“*Retinal Nerve Fiber Layer Area” (RNFLA). RNFLA was preferred over peripapillary retinal nerve fiber layer thickness measurements for studying the changes in the retinal nerve fiber layer due to the high variability of the latter in retinitis pigmentosa [19, 20]. The area between the internal limiting membrane and the outer plexiform layer was defined as the *“*Inner Retinal Area” (IRA). The outer plexiform layer and RPE delineated the *“*Outer Retinal Area” (ORA). Intraretinal cystic spaces were also outlined. Images were then analyzed using the in-build image analysis software of the device to determine the following spatial parameters:

1. Total area of the intraretinal cysts (CA), which is defined as the summation of the areas of all intraretinal fluid pockets
2. RNFLA
3. IRA
4. IRNA Inner retinal neuronal area (IRNA), which is defined as:
  IRNA = IRA-Inner Retinal Cyst Area-RNFLA
5. ORA
6. Total retinal area (TRA)
7. Intact outer limiting membrane length (iOLML)
8. Intact ellipsoid zone length (iEZL)
9. Optic nerve head diameter

If the cysts extend to ORA, the cyst area was excluded from the ORA calculation. Average values for each morphometric parameter from the three horizontal scans were used for further analyses. Correlations between the structural characteristics of the intraretinal cysts and anatomical features of the degenerating macula were sought to determine whether any anatomic features of the macular cysts can be predictive of the stage of the degenerative process at different layers of the retina. The functional impact of each morphometric parameter was also investigated by plotting them against the best-corrected logMAR visual acuity of the patients. Best-fit curves were derived using SigmaPlot 12.5 software (Systat Software, Inc., San Jose, CA, USA) to define the impact of retinal cysts on subjects’ visual acuities and previously listed retinal morphometric parameters. Values corresponding to the 75th, 50th, and 25th percentile of the effect were calculated using these curves.

### Statistical analyses

All quantitative data were expressed as mean ± standard error. The best-corrected visual acuity of the patients was converted to logMAR equivalents before statistical analysis. Statistical analyses were performed using SPSS statistics software (version 27.0, IBM Corp., USA). Differences in visual acuity and incidence of CME among different inheritance patterns were compared with one-way ANOVA. Intraobserver and interobserver (THT and AT) agreements in delineating the cysts’ borders and 8 retinal compartments were tested in randomly selected 10 OCT scans. Percent agreements and Kappa scores (κ) were used to express the consensus. Kappa scores of 0.61–0.80 were considered good agreement, and scores above 0.81 were accepted as excellent agreement. The associations between the intraretinal cyst area and subjects’ clinical and retinal morphometric features were tested using Generalized Estimating Equation with independent correlation matrix structure to account for inter-eye correlation by estimating the covariance among residuals from two eyes of a subject. Multivariate linear regression was also performed to evaluate the contribution of each variable to spatial parameters of the retinal cysts. Spatial correlations were corrected for age, refraction, and optic disc diameter. Total and inner retinal areas that included the areas covered by the retinal cysts were excluded from these analyses. Instead, outer retinal and inner retinal neuronal areas were used. A confidence level of p<0.05 was considered to be statistically significant.

## Results

The clinical records and OCT scans of 578 patients with retinitis pigmentosa were screened. 154 (25%) of the patients had CME. Among them, 103 patients (196 eyes; Right: 101; Left: 96) with retinitis pigmentosa were eligible for the study. The most common reason for the exclusion of the remaining 51 patients was vitreoretinal traction (n = 46), followed by retinal surgery (n = 3) and ocular hypertension (n = 2). The study cohort consisted of 53 female and 50 male patients. The mean age of the subjects was 45 ± 19 (7–76) years. Patients with X-linked retinitis pigmentosa (38 ± 10 years [27–50]) were significantly younger than the patients with autosomal dominant (43 ± 17 years [13–69]) and autosomal recessive (45 ± 18 years [13–76]) retinitis pigmentosa (p<0.001). The distribution of the subjects according to their genetic mutations and inheritance types of retinitis pigmentosa are given in Table 1. Forty-one (39.8%) subjects had autosomal dominant retinitis pigmentosa due to mutations in 9 different genes. Twenty-three other mutations were responsible for autosomal recessive retinitis pigmentosa in 58 (56.3%) subjects. Eight (7.8%) of the patients with autosomal recessive retinitis pigmentosa had syndromic retinitis pigmentosa as a part of Usher syndrome (5 patients with Usher 1, 1 patient with Usher 2, 2 patients with Usher 3). Four subjects (3.9%) had X-linked retinitis pigmentosa due to a mutation in the RPGR gene. The incidence of CME was significantly higher among screened patients with autosomal dominant retinitis pigmentosa (41/94; 43.6%, p<0.001), whereas patients with X-linked retinitis pigmentosa (4/94; 4.3%) had the lowest rate of CME (58/390; 14.9%; p<0.05).

**Table 1:** Causative genes and inheritance patterns of the patients.

Gene	Inheritance	Number of patients
RP1	ADRP	7
RHO	ADRP	9
KLHL7	ADRP	5
PRPH2/RDS	ADRP	3
IMPDH1	ADRP	4
PRPF31	ADRP	3
NRL	ADRP	3
IMPG2	ADRP	3
PDE6B	ADRP	4
C2orf71	ARRP	2
IFT172	ARRP	3
ARMC9	ARRP	2
USH2A	ARRP	6
LCA5	ARRP	3
VCAN	ARRP	3
GUCA1a	ARRP	3
CNGB1	ARRP	4
COQ2	ARRP	2
CEP290	ARRP	3
MAK	ARRP	4
DHDDS	ARRP	3
REEP6	ARRP	2
CRB1	ARRP	2
EYS	ARRP	4
PDSS1	ARRP	2
NPHP1	ARRP	2
TTLL5	ARRP	2
KIAA1549	ARRP	1
ACBD5	ARRP	1
RPGR	XLRP	4
MYO7A	ARRP	4
PCDH15	ARRP	1
CLRN1	ARRP	3

ADRP = Autosomal dominant retinitis pigmentosa (n = 41)

ARRP = Autosomal recessive retinitis pigmentosa (n = 58)

XLRP = X-linked retinitis pigmentosa n = 4)

The mean best-corrected visual acuity of the enrolled subjects was 20/44 ± 20/39 (20/20-20/800). Clinical exams and OCT scans confirmed the presence of bilateral CME in 93 (90.3%) subjects, whereas the remaining 10 (9.7%) subjects had CME only in one eye. In all eyes with CME, intraretinal cysts were present in the inner nuclear layer. In 48 (24.5%) eyes, cystic spaces extended into the outer nuclear layer.

**Fig 2:**
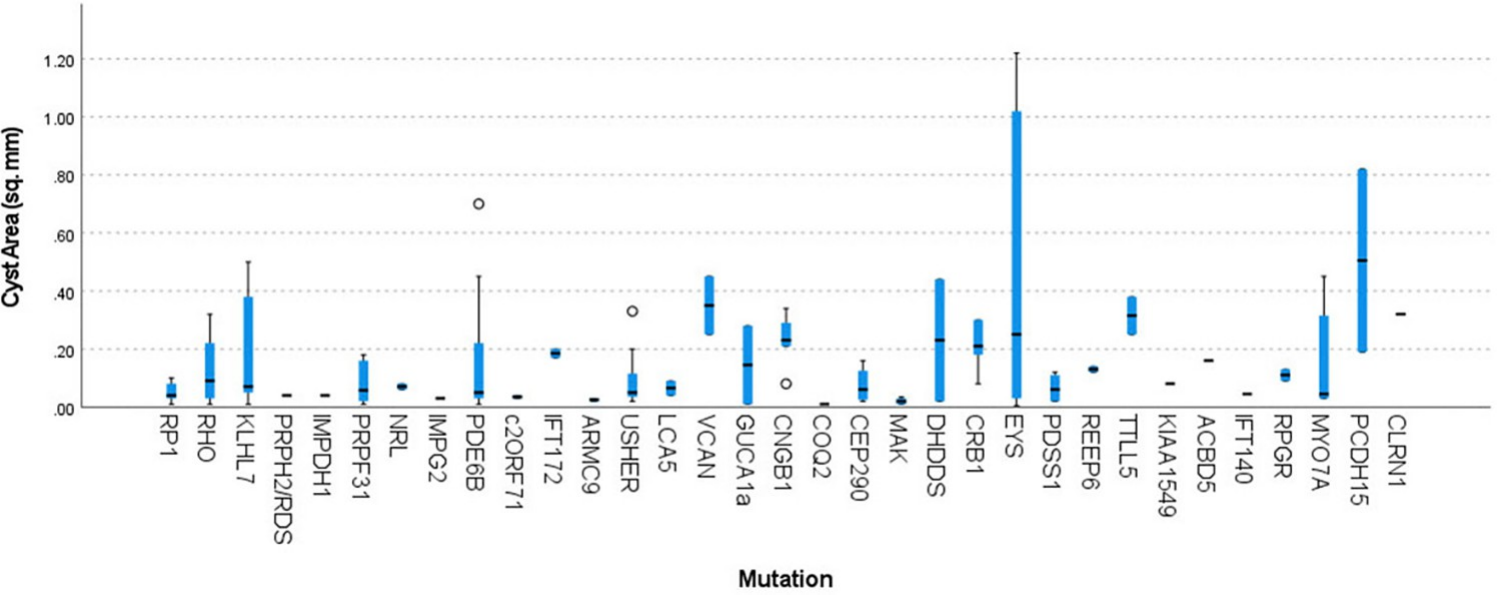
Distribution of intraretinal cyst sizes among underlying mutations causing retinitis pigmentosa. Comparison of cyst sizes in subjects with different underlying genetic mutations did not reveal any differences (p = 0.08).

The interobserver agreement in the morphometric delineation of the borders of the cysts and retinal compartments size was 0.96 (95% CI: 0.87–0.98) with a κ score of 0.94 (95% CI: 0.88–0.99). The intraobserver agreement on morphometric measurements were 0.98 (95% CI: 0.92–0.99) and 0.98 (95% CI: 0.90–0.99) with corresponding κ scores of 0.99 and 0.98. Total intraretinal cyst area was 0.14 ± 0.18 mm^2^ (0.01–1.22 mm^2^, Table 2). All morphometric parameters were exhibiting significant positive correlations with the cyst area, including RNFLA (0.35 ± 0.11 mm^2^ [0.12–0.83], r = 0.266, p = 0.001), IRNA (1.06 ± 0.22 mm^2^ [0.49–1.64], r = 0.197, p = 0.009), IRA (1.53 ± 0.33 mm^2^ [0.77–2.87], r = 0.647, p < 0.001), ORA (0.57 ± 0.19 mm^2^ [0.11–1.06], r = 0.272, p = 0.0005), TRA (2.10 ± 0.41 mm^2^ [0.99–3.66], r = 0.637, p < 0.001), iOLML (3416.1 ± 1513.7 mm [307–7739], r = 0.218, p = 0.004) and iEZL (2402.2 ± 1413.5 mm [128–7625], r = 0.180, p = 0.016). A negative correlation was found between the cyst area and the patient’s age (r = -0.266, p < 0.001). Patient’s gender (p = 0.49), inheritance pattern (p = 0.16) and the underlying genetic mutation had no effect on the size of the intraretinal cysts (p = 0.08; Fig 2).

**Table 2:** Correlations of anatomical and functional parameters.

Anatomical Structure	Size	^a^Correlation with retinal cyst size	^a^Correlation with visual acuity
Cyst Area (mm2)	0.14 ± 0.18 (0.01–1.22)	-	0.063 (P = 0.227)
Retinal Nerve Fiber Layer Area (mm2)	0.35 ± 0.11 (0.12–0.83)	0.266 (P = 0.001)	0.325 (P < 0.001)
^b^Inner Retinal Neuronal Area (mm2)	1.06 ± 0.22 (0.49–1.64)	0.197 (P = 0.009)	0.160 (P = 0.028)
Inner Retinal Area (mm2)	1.53 ± 0.33 (0.77–2.87)	0.647 (P < 0.0001)	0.238 (P = 0.002)
^c^Outer Retinal Area (mm^2^)	0.57 ± 0.19 (0.11–1.06)	0.272 (P < 0.001)	- 0.072 (P = 0.197)
Total Retinal Area (mm^2^)	2.10 ± 0.41 (0.99–3.66)	0.637 (P < 0.0001)	0.159 (P = 0.029)
Intact Outer Limiting Membrane (mm)	3416.1 ± 1513.7 (307–7739)	0.218 (P = 0.004)	- 0.193 (P = 0.010)
Intact Ellipsoid Zone (mm)	2402.2 ± 1413.5 (128–7625)	0.180 (P = 0.016)	- 0.296 (P < 0.001)

^a^Pearson Product-Moment Correlation

^b^Inner Retinal Neuronal Area = Area within the inner retina not covered by RNFL and cysts

^c^Outer Retinal Area = Area of cysts within the outer retinal zone were excluded

**Fig 3:**
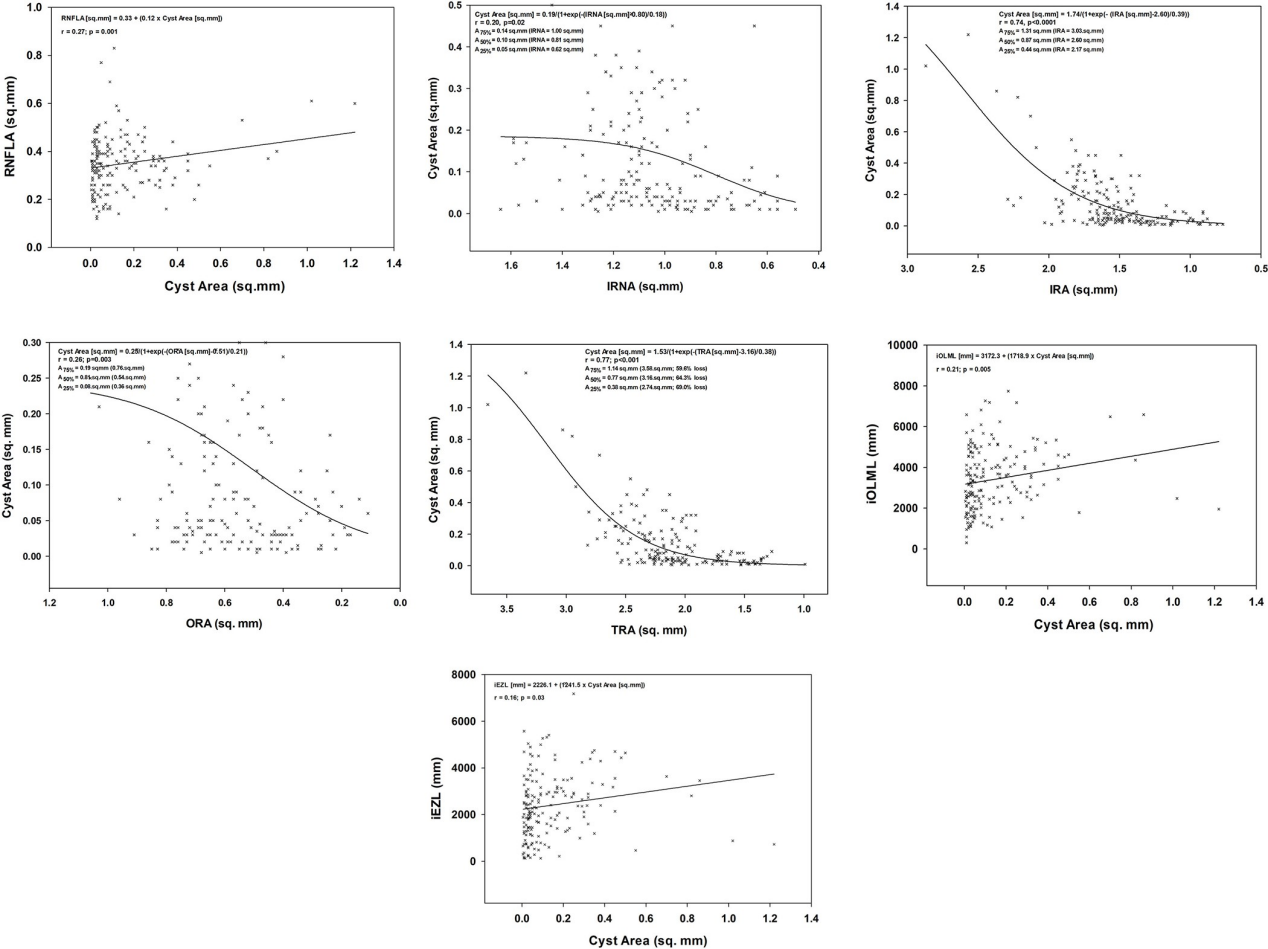
Best-fit curves defining the relationship between cyst size and other retinal anatomical parameters. **(A)** A significant linear correlation exists between the size of the intraretinal cysts and RNFLA (r = 0.27, p = 0.001), indicating that RNFL becomes thickened at times when intraretinal cysts are at the peak of their size. **(B)** The change in retinal cyst size and the IRNA can be best expressed with a 3-parameter Sigmoidal curve in the form of f = a/(1+exp(-(x-x0)/b)). Retinal cysts resolve as the IRNA decreases in the late phases of the degenerative process. (r = 0.20, p = 0.02). **(C)** Intraretinal cysts diminish with the thinning of the inner retina (r = 0.74, p < 0.001). The high correlation is mostly due to the presence of cysts in the inner nuclear layer. **(D)** Intraretinal cysts are the biggest in the early phases of the disease. As outer retinal retina degenerates, the size of the cysts gets smaller (r = 0.26, p = 0.003). **(E)** Resolution of intraretinal cysts coincides with decrease in total retinal area (r = 0.77, p < 0.001). **(F)** There is a positive linear correlation between cyst size and remaining OLM, indicating that cysts develop early in retinal degeneration and fade out as the outer retina degenerates (r = 0.21; p = 0.005). **(G)** Like iOLML, iEZL positively correlates with the cyst area, indicating that degeneration of the photoreceptors is coupled with the resolution of the cysts (r = 0.16; p = 0.03).

Multivariate linear regression analyses revealed ORA (B = 0.214; p = 0.008), age (B = -0.003; p < 0.001), and RNFLA (B = 0.411; p = 0.005) can explain 19.0% of the variance of the retinal cyst sizes (r = 0.44; F = 10.96; p < 0.001). Best-fit curves confirmed that there is a linear correlation between the size of the intraretinal cysts and RNFLA, indicating thickening of the RNFL in the presence of bigger cysts (r = 0.27, p = 0.001, Fig 3A). Similar linear relationships were observed between the cyst size and the remaining iOLML (r = 0.21, p = 0.005, Fig 3F) and iEZL (r = 0.16, p = 0.03, Fig 3G). Correlation between the retinal cysts and IRNA (r = 0.20, p = 0.02, Fig 3B), IRA (r = 0.74, p < 0.001, Fig 3C), ORA (r = 0.26, p = 0.003, Fig 3D), and TRA (r = 0.77, p < 0.001, Fig 3E) best-fit to a 3-parameter Sigmoidal curve in the form of f = a/(1+exp(-(x-x0)/b)). All these curves indicate that cyst gets smaller in cases in cases with advanced outer retinal degeneration.

**Fig 4:**
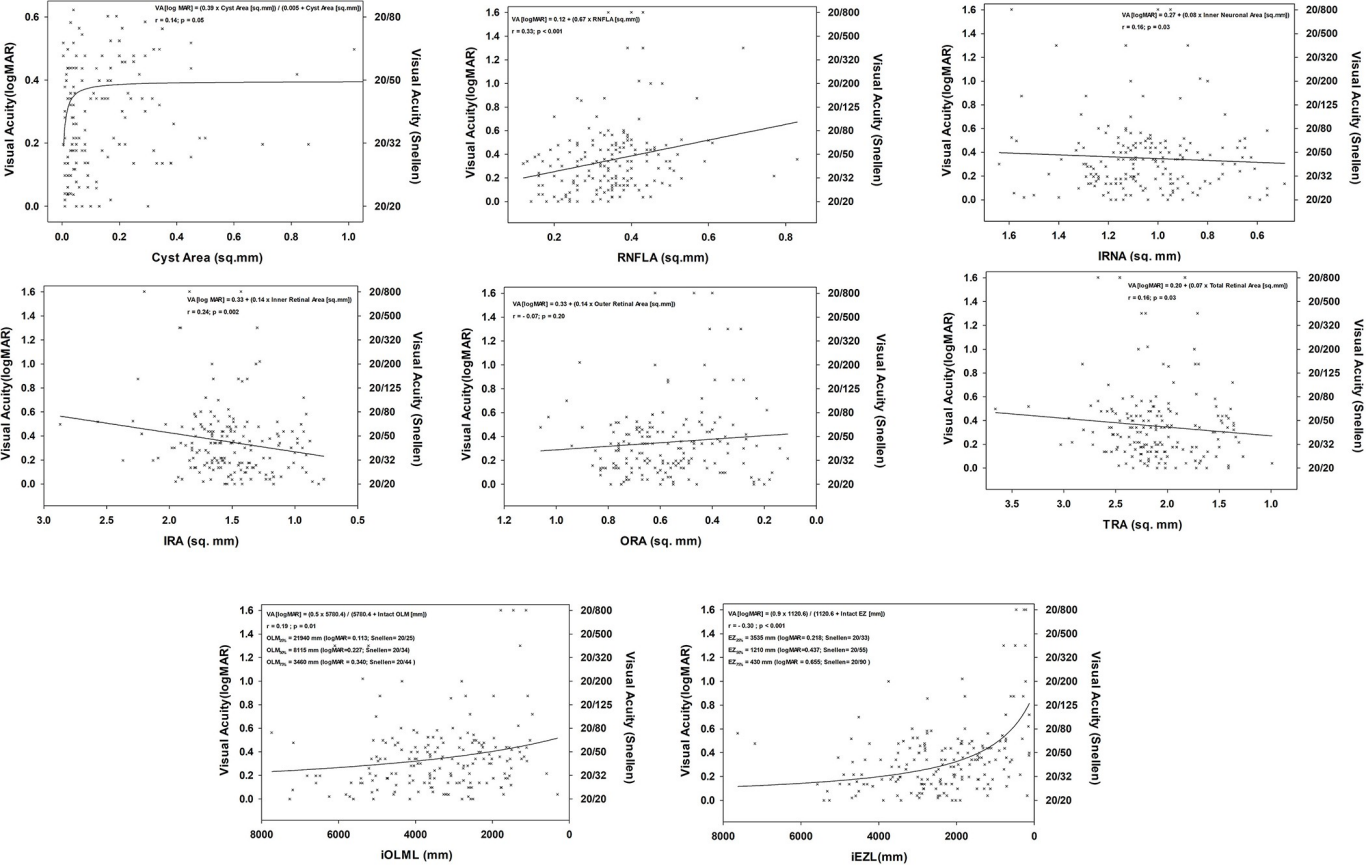
Impact of change in anatomical features on visual acuity. **(A)** A 2-parameter single rectangular hyperbola best-fits to explain the impact of retinal cysts on visual acuity (r = 0.14, p = 0.05). Cysts cause a 2-line drop in visual acuity early during development. Although cysts may enlarge in time, their impact on visual acuity remains the same. **(B)** RNFLA has the highest linear correlation with logMAR visual acuity among other studied parameters (r = 0.325, p < 0.001). **(c)** A weak linear correlation existed between the IRNA and visual acuity (r = 0.160, p = 0.028). **(D)** An increase in IRA with the development of cysts decreases the patients’ visual acuity (r = 0.238, p = 0.002). **(E)** No significant correlation was detected between ORA and visual acuity (r = - 0.072, p = 0.197). **(F)** Similar to the relationship of visual acuity with IRA, the size of cysts affects both the TRA and visual acuity (r = 0.159, p = 0.029). Patients have better visual acuity if the cysts are small; as they get bigger, their visual acuity worsens, and TRA increases. **(G)** A significant correlation exists between the OLM and visual acuity, which fits-best to a 2-parameter hyperbolic curve in the form f = (a*b)/(b+x) (r = 0.19, p = 0.01). The 50^th^ percentile of this effect corresponds to 8115 mm of intact OLM and visual acuity of 20/34. **(H)** iEZL significantly correlates with visual acuity. Distribution of the data best-fits to a 2-parameter hyperbolic curve in the form f = (a*b)/(b+x) (r = 0.30, p<0.001). The 50^th^ percentile of this effect corresponds to 1210 mm of intact EZ and visual acuity of 20/55.

There were significant differences in the mean visual acuities of subjects with different inheritance patterns (p = 0.02). Subjects with autosomal dominant retinitis pigmentosa have the best visual acuity 0.23 ± 0.18 logMAR (Snellen equivalent: 20/34), followed by subjects with autosomal recessive 0.36 ± 0.29 logMAR (Snellen equivalent: 20/46) and X-linked forms 1.08 ± 0.30 logMAR (Snellen equivalent: 20/240). No significant correlation was detected between the best-corrected visual acuity and patient’s age (r = 0.112; p = 0.091), gender (r = 0.025; p = 0.382), and ORA (r = - 0.072, p = 0.197, Fig 4E, Table 2). Analysis indicated that the impact of cyst size on visual acuity could be best defined with a 2-parameter single rectangular hyperbolic curve in the form of f = a*x/(b+x) (r = 0.14, p = 0.05, Fig 4A). This best-fit curve indicates that the development of the cysts can cause a two-line decrease in visual acuity early in the development of the cysts, which becomes stable afterward.

All other morphometric parameters exhibited significant correlations with visual acuity including RNFLA (r = 0.325, p < 0.001, Fig 4B), IRNA (r = 0.160, p = 0.028, Fig 4C), IRA (r = 0.238, p = 0.002, Fig 4D), TRA (r = 0.159, p = 0.029, Fig 4F), iOLML (r = - 0.193, p = 0.01, Fig 4G), and iEZL (r = - 0.296, p < 0.001, Fig 4H). Multivariate linear regression analyses indicated iEZL (B = -5.16E-5; p < 0.001), the inheritance pattern (B = 0.04; p = 0.028), and RNFLA (B = 0.751; p< 0.001) as the main variables that can determine the 20.3% of the visual acuity variance in the setting of CME in retinitis pigmentosa (r = 0.45; F = 4.94; p = 0.028).

## Discussion

This study demonstrates that CME is expected, with a prevalence of around 25% in patients with retinitis pigmentosa. This proportion falls within the limits of previously reported rates of CME in different studies [21, 22]. In conjunction with previous reports [23, 24], our cohort demonstrated a higher incidence of CME among patients with autosomal dominant inheritance than other inheritance patterns. We did not find any significant difference in the cyst area among different inheritance patterns and gene mutations, suggesting that common secondary pathogenetic mechanisms, rather than the underlying genetic mutations, are the primary driving factor for the development of CME. We observed that the retinal anatomical changes correlate with the size and resolution of the intraretinal cysts during the retinal degeneration process. Our analyses revealed that larger intraretinal cysts are associated with higher ORA, RNFLA and younger age. These three factors could explain 19.0% of the variance in cyst size in our cohort. It is not surprising that ORA impacts retinal cyst size, considering that retinitis pigmentosa is primarily an outer retinal degeneration. All current theories about CME pathogenesis in retinitis pigmentosa converge on outer retinal degeneration as a trigger for the secondary events leading to CME, such as the breakdown of the blood-retina barrier [25, 26], Müller cell dysfunction, disruption of subretinal ionic hemostasis [27], or induction of an autoimmune response to sequestrating retinal antigens [17]. A larger ORA naturally translates to more photoreceptors degenerating, amplifying the impact of events leading to cystoid macular degeneration. Similarly, younger patients with more photoreceptors to degenerate [28] are expected to develop larger retinal cysts.

Varying retinal nerve fiber layer thicknesses have been reported in patients with retinitis pigmentosa [14, 20, 29]. The focal nature of the retinal nerve fiber layer defects [30]. variability of the results [31], and the differences in employed methodology [19] can explain the equivocal results. To overcome these difficulties, we preferred calculating the RNFLA rather than peripapillary retinal nerve fiber layer thickness. This method reflects any CME-induced focal changes in retinal nerve fiber layer thickness, which can be missed by studying the nerve fiber layer thickness only at points along the perimeter of a peripapillary circle. The strong correlations we observed between RNFLA, the cyst area, and visual acuity suggest that intraretinal cysts further deteriorate a patient’s vision by compressing the retinal nerve fiber layer and leading to axonal stasis, similar to what we reported in diabetic macular edema and retinal vein occlusion [32]. Axonal compression can also be the underlying cause of electrophysiological dysfunction [33] and histologic evidence of ganglion cell death [34, 35] in retinitis pigmentosa with CME. Initial thickening and subsequent thinning of the RFNL with the progression of outer retinal degeneration was reported earlier [29, 36, 37]. The preferential nature of this change in the RNFL temporal to the disc [37, 38] supports our view about the potential impact of CME on RNFL thickness. RNFLA has the most significant impact on visual outcomes in the presence of CME (B = 0.751). Best-fit curves indicate that a 3.5 times increase in the RNFLA can result in an average of 15 letters of vision loss. (Fig 4B) We previously used a mathematical model to estimate the pressure exerted by intraretinal cysts on surrounding retinal tissue [32]. Applying the same model to our current cohort, we may estimate that retinal cysts with an average area of 0.14 ± 0.18 mm^2^ and a radius of 0.21 ± 0.24 mm may exert a force on the surrounding retinal tissue comparable to an increase in intraocular pressure up to 77 mmHg. Cysts in other body parts are measured to generate forces at this magnitude [39, 40]. Axoplasmic flow within retinal axons will be disturbed upon exposure to high local pressure or stretching due to axonal compression [41]. [41] There may be several other causes for the thickening of the RNFLA in retinitis pigmentosa, including the stagnation of the axoplasmic flow due to reactive gliosis or altered retinal vascular physiology. However, until the exact mechanism of RNFL thickening is further elucidated, early recognition and treatment of CME remain reasonable precautionary measures for relieving the axonal compression and preserving the remaining visual field.

Similar to a previous study [42], we observed a reduction in cyst size in eyes with advanced stages of retinal degeneration as evidenced by a decrease in all studied morphometric parameters. Although thinning of the ORA seems to be directly linked to CME size, the predominant location of the cysts in the inner nuclear layer suggests Müller cell dysfunction as the leading cause of CME in retinitis pigmentosa. The impact of photoreceptor cell degradation products on Müller cell expression of aquaporin-4 and inwardly rectifying potassium channels (Kir4.1) has been thought to be responsible for altered fluid transport function and, thus, CME [27]. Muller cell activation in retinitis pigmentosa [43] can also alter the expression of these molecules [44] and disrupt the potassium and water transport functions.

The impact of CME on visual acuity was limited on average to 2-lines of loss early in its development (Fig 4A). After that, outer retinal degeneration and several other epiphenomena took precedence in visual loss, and the impact of cysts on visual acuity remains the same. The limited effect of the CME size on the visual acuity of patients with retinitis pigmentosa [45, 46] remains in contrast to the impact of CME seen in patients with diabetic macular edema and branch retinal vein occlusion [47]. Several morphometric parameters indicating retinal atrophy were correlated with decreases in visual acuity, such as IRNA, IRA, TRA, iOLML, and iEZL [48–50]. However, ORA failed to correlate with visual acuity. Failure to correlate outer retinal spatial features with the functional status of the retinitis pigmentosa patient was reported earlier [51, 52]. This paradoxical finding can result from the false expansion of the outer retina by Müller cell invasion [34] or simply due to a discrepancy created by the cone photoreceptor cell dormancy [53]. Our analyses revealed that iEZL, the inheritance pattern, and RNFLA play a role in 20.3% of the visual acuity variance in the setting of CME in retinitis pigmentosa. iEZL has previously been a reliable indicator of patients’ visual acuity and residual visual field in retinitis pigmentosa [54, 55]. Patients with autosomal dominant retinitis pigmentosa tend to have a milder course and thus have relatively healthier photoreceptors that have the best visual acuity. In contrast, patients with the X-linked recessive form of retinitis pigmentosa often present with the most severe loss of photoreceptors and have the worst visual acuity.

We believe our study is unique due to its methodology and the inclusion of the highest number of genotyped retinitis pigmentosa patients with CME. However, the findings of our study must be interpreted with several limitations in mind. The first is that this is a retrospective cross-sectional study and carries forward all inherited weaknesses. It is essential to verify the correlations between the anatomical and functional parameters and their temporal changes with long-term follow-up analyses of patients. Second, these significant correlations also may not necessarily imply direct causation. The pathogenesis of CME in retinitis pigmentosa probably involves complex associations arising from multiple interactions. Although anatomical changes in different retinal compartments significantly correlate with intraretinal cyst size, such changes alone may not be enough to explain the CME pathogenesis. It is obvious that events leading to changes in the anatomical features of these compartments affect each other and contribute to the evolution and resorption of the intraretinal cysts. An appropriately designed experiment will be required to determine the interaction between these anatomical layers. However, knowing the association of cysts’ size with changes in different retinal layers may help clinicians manage CME in retinitis pigmentosa. Third, confounding factors might have impacted measured parameters, such as other systemic and ocular co-morbidities. To overcome this difficulty, we applied strict criteria to exclude several conditions that may interfere with measured parameters.

In summary, CME is a relatively common finding in patients with retinitis pigmentosa. Morphometric analysis of retinal layers and their correlation with spatial properties of the intraretinal cysts suggest a high negative correlation between the outer retinal degeneration and the size of the cysts. The thickening of the RNFL may be explained by the compressive forces generated by the intraretinal cysts, resulting in axonal stasis. The presence and impact of CME may often be overlooked in retinitis pigmentosa due to the plethora of clinical findings and causes of visual loss. Compressional atrophy of the nerve fiber layer and permanent deterioration of central vision can severely impact patients’ quality of life, especially considering that most patients with retinitis pigmentosa already have peripheral visual field loss. Thus, care must be taken to recognize and treat CME early during the development of retinitis pigmentosa.

## Abbreviations

CME: Cystoid Macular Edema
RPE: Retinal Pigment epithelium
RNFLA: Retinal Nerve Fiber Layer Area
IRA: Inner Retinal Area
IRNA: Inner Retinal Neuronal Area
ORA: Outer Retinal Area
TRA: Total Retinal Area
iOLML: Intact Outer Limiting Membrane Length
iEZL: Intact Ellipsoid Zone Length
OCT: Optical Coherence Tomography
ILM: Inner Limiting Membrane
OPL: Outer Plexiform Layer
EZ: Ellipsoid zone
OLM: Outer Limiting membrane
